# Tri-State Medical Association

**Published:** 1883-07-20

**Authors:** 


### *TRI-STATE MEDICAL ASSOCIATION.*

In a note from the President we learn the ninth annual meeting of the Tri-State Medical Association will be held in Indianapolis, September 18th, 19th and 20th. The work is already far advanced, and the title of each paper should be sent in at once. Papers must not exceed 25 minutes. It is also the rule that each physician who registers must be a member of a local or State Society in good repute. All such are invited.

Notice of papers or cases to be presented may be sent to the chairman of the committee on programme, Dr. J. L. Thompson, Indianapolis; to the Secretary, Dr. G. W. Burton, Mitchell, Indiana, or to the President, Dr. Wm. Porter, St. Louis.

